# Detection of Antimicrobial Residues in Poultry Litter: Monitoring a Risk through a Selective and Sensitive HPLC–MS/MS Method

**DOI:** 10.3390/ani11051399

**Published:** 2021-05-14

**Authors:** Karina Yévenes, Ekaterina Pokrant, Lina Trincado, Lisette Lapierre, Nicolás Galarce, Betty San Martín, Aldo Maddaleno, Héctor Hidalgo, Javiera Cornejo

**Affiliations:** 1 Department of Preventive Animal Medicine, Faculty of Veterinary and Animal Sciences, University of Chile, Santiago 8820808, Chile; kariyevenescoa@gmail.com (K.Y.); katiavalerievna@ug.uchile.cl (E.P.); lina.trincado@ug.uchile.cl (L.T.); llapierre@uchile.cl (L.L.); ngalarce@ug.uchile.cl (N.G.); 2 Programa de Doctorado en Ciencias Silvoagropecuarias y Veterinarias, Campus Sur Universidad de Chile, Santiago 8820808, Chile; amaddaleno@veterinaria.uchile.cl; 3 Laboratory of Veterinary Pharmacology (FARMAVET), Faculty of Veterinary and Animal Sciences, University of Chile, Santiago 8820808, Chile; bsmartin@uchile.cl; 4 Laboratory of Avian Pathology, Faculty of Veterinary and Animal Sciences, University of Chile, Santiago 8820808, Chile; hhidalgo@uchile.cl

**Keywords:** antimicrobials, poultry litter, agriculture fertilizer, HPLC–MS/MS, validation

## Abstract

**Simple Summary:**

After administration of antimicrobials, poultry excrete significant concentrations of antimicrobials through their droppings, which accumulate in the litter where poultry are housed. Poultry litter, which consists mainly of wood shavings, feathers, and droppings, is widely used as an agricultural fertilizer worldwide. The period that antimicrobials persist in agricultural soils could present various environmental and public health concerns. Thus, in this research, a method to identify different families of antimicrobials in poultry litter was developed. Results show that HPLC–MS/MS can reliably detect nine different compounds from three families of antimicrobials. This method was used to identify antimicrobials from commercial poultry flocks, providing a valuable and specific tool to monitor these residues in poultry litter prior to its use as an agricultural fertilizer.

**Abstract:**

Tetracyclines, sulphonamides, and quinolones are families of antimicrobials (AMs) widely used in the poultry industry and can excrete up to 90% of AMs administrated, which accumulate in poultry litter. Worryingly, poultry litter is widely used as an agriculture fertilizer, contributing to the spread AMs residues in the environment. The aim of this research was to develop a method that could simultaneously identify and quantify three AMs families in poultry litter by high-performance liquid chromatography–tandem mass spectrometry (HPLC–MS/MS). Samples of AMs free poultry litter were used to validate the method according to 657/2002/EC and VICH GL49. Results indicate that limit of detection (LOD) ranged from 8.95 to 20.86 μg kg^−1^, while limits of quantitation (LOQ) values were between 26.85 and 62.58 µg kg^−1^ of tetracycline, 4-epi-tetracycline, oxytetracycline, 4-epi-oxytetracycline, enrofloxacin, ciprofloxacin, flumequine, sulfachloropyridazine, and sulfadiazine. Recoveries obtained ranged from 93 to 108%. The analysis of field samples obtained from seven commercial poultry flocks confirmed the adequacy of the method since it detected means concentrations ranging from 20 to 10,364 μg kg^−1^. This provides us an accurate and reliable tool to monitor AMs residues in poultry litter and control its use as agricultural fertilizer.

## 1. Introduction

The use of antimicrobials (AMs) in poultry production dates to the 1940s when it became an essential tool in the development of the poultry industry. AM administration was essential because infectious diseases of bacterial origin in broiler flocks can account for 20% of gross production value losses [1]. AM use has become significant with more than three million kilograms of AMs used in the poultry industry in the United States in 2016 [2]. Moreover, it is estimated that the global annual average consumption of AMs by poultry is 148 mg kg^−1^ [3]. Tetracyclines, sulphonamides, and quinolones are one of the AMs administered to poultry flocks [4,5]. Additionally, in Chile, there are pharmaceutical formulations of these AMs register for use in poultry production [6,7].

Poultry can excrete between 45 and 96% of AMs through their droppings as active metabolites or in their original form [8,9]. The AMs excreted accumulate in poultry litter, the principal by-product of the poultry industry. Poultry litter is a heterogeneous compound, consisting mainly of poultry droppings, litter material (e.g., wood shavings), dead skin, food debris, water, microbiota, and poultry feathers [10,11,12]. Moreover, concentrations reaching 2947 μg kg^−1^ of sulfachloropyridazine and oxytetracycline have been detected on poultry feathers, further contributing to the accumulation of AMs in litter [13,14]. Several families of AMs have already been identified in poultry litter in concentrations ranging from 0.01 to 152 mg kg^−1^ [15,16,17]. Moreover, currently, there are no regulations concerning the tolerance limits for residues of AMs in by-products generated by the animal industry, and the maximum residue limits (MRL) are only applicable to edible animal tissues [18].

The main problem is that poultry litter containing AM residues is widely used by farmers around the world as agricultural fertilizer. For centuries, litter has been applied to land to increase crop yield, due to the presence of nitrogen, phosphorus, and potassium [19,20,21,22]. This practice has been described as one of the main pathways by which AMs enter the environment [4,21,23]. In this context, Zhang et al. [24] identified more than 15 AMs, in concentrations ranging from 0.1 to 333.5 μg kg^−1^ in agricultural soils that were consistently amended with broiler poultry manure. Similarly, Wei et al. [25] analyzed 27 soil samples fertilized with broiler poultry droppings and detected concentration of tetracycline, quinolones, and sulphonamides up to 30,779, 5305, and 1316 μg kg^−1^ of soil analyzed, respectively. Hou et al. [26] conducted similar research in northern China, where tetracyclines, chlortetracycline, oxytetracycline, and doxycycline were the AMs detected at higher concentrations. The use of fertilizer obtained from farm animal feces, specifically poultry litter, contributes to the dissemination of AMs into the environment.

The AMs in agricultural soil can persist for days to years [27] and bioaccumulate in cultivated vegetables [28] or migrate as a result of runoff toward new water bodies [29]. The presence of AMs in soil, water, and vegetables may have an adverse effect on human health, such as hypersensitivity, toxicity, and disruption of the gut microbiota [30,31,32,33] and contribute to the development of antimicrobial resistance [34]. Therefore, the presence of AM residues in the environment needs to be evaluated in-depth [35].

Previous works demonstrate the importance of efficiently and effectively identifying AMs in poultry litter. Multiple AMs have been identified by high-performance liquid chromatography-tandem mass spectrometry (HPLC–MS/MS) in matrices other than poultry litter [36,37,38]. Nevertheless, methods for poultry litter are limited [15] because the physical and chemical properties of both the poultry litter and various AMs adds to the complexity of the method. The aim of this research was to develop a specific method able to detect and quantify different families of AMs (tetracyclines, sulphonamides, and quinolones) and their main active metabolites in poultry litter. Analysis prior to its use as an agricultural fertilizer could help to prevent environmental contamination and the resulting risks for public health. 

## 2. Materials and Methods

### 2.1. Sample Collection

Poultry litter was obtained from broiler chicken, genetic line Ross^®^ 308 (Aviagen Inc., Huntsville, AL, USA). Animals were not treated with AMs and managed following the recommendations described in the “Poultry Industry Manual of the United States Department of Agriculture” to simulate production farming [39]. Per 1.5 m^2^ of pen, 10 animals were placed on a smooth floor with 10 cm of wood shavings. Water, food, and wood shavings present in the litter were analyzed prior to exposure to birds by HPLC–MS/MS to ensure no presence of AM residues.

Poultry breeding was performed in compliance with Chilean Law No. 20,380 “On the protection of animals “ [40] and the Institutional Committee for the Care and Use of Animals (CICUA, as per initials in Spanish) of the University of Chile issued a certificate (permit code 18187-VET-UCH-E1). Furthermore, the Directive 2010/63/EU “On the protection of animals used for scientific purposes” [41] and the Regulation (CE) No. 1099/2009) “On the protection of animals at the time of slaughter” were respected [42]. 

**Figure 1:**
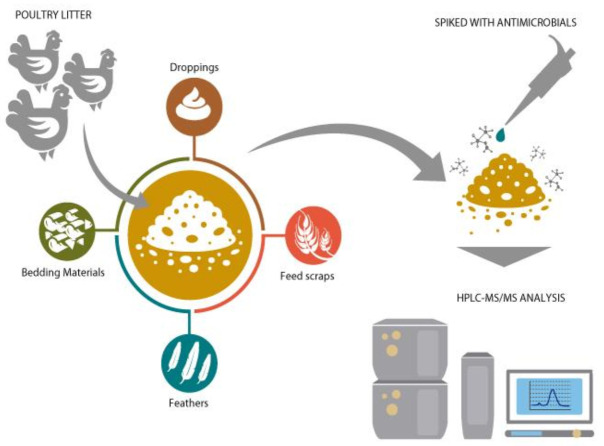
Production, spiked, and analysis of poultry litter.

After 42 days of rearing, poultry litter was collected, consisting mainly of droppings, feathers, food, feed scraps, and bedding material, stored in plastic bags, and placed in a freezer at −20 °C until it was used as a matrix spiked with AM residues and analyzed by HPLC–MS/MS (Figure 1).

### 2.2. Standard, Reagents, and Chemicals

Tetracycline (TC), 4-epi-tetracycline (4-epi-TC), oxytetracycline (OTC), 4-epi-oxytetracycline (4-epi-OTC), enrofloxacin (EFX), ciprofloxacin (CFX), flumequine (FLU), sulfachloropyridazine (SCP), and sulfadiazine (SDZ) with certified purity (>90%) were manufactured by Sigma Aldrich, Inc. (Merck KGaA, Darmstadt, Germany). The internal standard (I.S.) enrrofloxacin-D5 (EFX D5), sulfamethazine-phenyl-13C6 (SMZ 13C6), and tetracycline-D6 (TC D6) with certified purity (>95%) were manufactured by Toronto Research Chemicals (Toronto, ON, Canada). 

Stock solutions of TC, 4-epi-TC, OTC, 4-epi-OTC, EFX, CFX, FLU, SCP, and SDZ were prepared in methanol at a concentration of 1000 µg mL^−1^ and stored at −80 °C. Intermediate or working solutions were prepared using stock solutions diluted with methanol at a concentration of 1000 ng mL^−1^ which was stored at −80 °C.

McIlvaine–EDTA buffer (0.1 M, pH 4.0 ± 0.1), used for analyte extraction, was prepared by mixing 280 mL of solution A (14.2 g of hydrogen phosphate dihydrate in 500 mL of water), with 500 mL of solution B (10.5 g of citric acid monohydrate in 500 mL of water), then 74.4 g of EDTA was added. Milli-Q^®^ water was added to a final volume of 2 L. All other reagents, such as water, methanol, and acetonitrile were of analytical grade and were manufactured by Fisher (Thermo Fisher Scientific, Waltham, MA, USA) or Merck (Merck KGaA, Darmstadt, Germany).

### 2.3. Extraction of Antimicrobials

Poultry litter samples were processed in a SKYMSEN^®^ (Brusque, Brazil) grinder to homogenize the sample and reduce the size of the constituent. 

For the analysis, 1 ± 0.01 g of litter was placed in a 50 mL polypropylene tube and then spiked with different and equidistant analytes concentrations and internal standard described in Section 2.5. The extraction of analytes was carried out 15 min afterward. The samples were spiked with 8 mL of McIlvaine–EDTA buffer (0.1 M, pH 4.0) and 2 mL of acetonitrile. The tube was then shaken, sonicated, and centrifuged in a Hettich^®^ ROTOFIX 32A (Hettich Lab Technology, Beverly, MA, USA) at 2.700 g for 10 min. The supernatants obtained were passed through Whatman™ filters grade GF/A (1.6 µm) (Merck KGaA, Darmstadt, Germany) and transferred to another 50 mL polypropylene tube. Then, the samples were diluted by adding 13 mL of McIlvaine–EDTA buffer and shaken and centrifuged for 5 min at 2700 g. At the same time, solid-phase extraction columns SPE Supel™ Select HLB (Supelco, MERCK KGaA, Darmstadt, Germany) were conditioned with 5 mL of methanol and 5 mL of HPLC-grade water. Samples were passed through the columns, washed with 5 mL of HPLC-grade water, and dried with a manifold pump for 5 min. The samples were eluted with 10 mL of methanol and evaporated under nitrogen flow in a water bath at 45 ± 5 °C. The samples were reconstituted with 200 μL of methanol and 300 μL of HPLC-grade water and then shaken, sonicated, and centrifuged for 5 min at 2700 g. The upper phase of the samples was transferred to Eppendorf microtubes, which were centrifuged in a VWR^®^ 2417R (Avantor, Radnor, PA, USA) at 17,136 g for 10 min. Finally, the supernatants were transferred through 33 mm Millex^®^ filters (Merck KGaA, Burlington, MA, USA) into glass vials for subsequent analysis by HPLC–MS/MS.

### 2.4. Instrument Analysis 

Samples were analyzed using an Agilent^®^ 1290 series liquid chromatograph (Agilent Technologies, Santa Clara, CA, USA), coupled with an ABSCIEX^®^ API 5500 mass spectrometer (SCIEX, Framingham, MA, USA), and a SunfireTM C18 chromatographic column (Waters Corp., Milford, MA, USA) of 3.5 μm and 150 × 2.1 mm was used. The analytes were separated chromatographically using a mobile gradient of 0.1% formic acid in water (Phase A) and 0.1% formic acid in methanol (Phase B). The flow rate was adjusted to 0.2 mL/min, the injection volume was 20 µL, the duration was 25.423 min, and the column temperature was set at 35 °C ± 1 °C. The liquid chromatographic pump gradient is shown in Table 1. The scans per peak are shown in Table S1.

The criteria to identify the different AMs and their active metabolites was the monitoring of the masses of the precursor and fragment ions (Table S2). In addition, different parameters were used for the operation of the mass detector (Table 2). The chromatographic integration of the samples was performed using Analyst^®^ software version 1.6.2 (SCIEX, Framingham, MA, USA).

Samples were processed and analyzed at the Veterinary Pharmacology Laboratory (FARMAVET, as per initials in Spanish) of the Faculty of Veterinary and Animal Sciences at the University of Chile, which is accredited under ISO 17,025 standards.

### 2.5. Method Validation Procedure

To assure that the present method was suitable for detecting and quantifying AM residues in poultry litter, several performance parameters such as analyte retention time (RT), limit of detection (LOD), limit of quantification (LOQ), recovery, precision (through repeatability and intra-laboratory reproducibility) and linearity of the calibration curve were evaluated. For this purpose, an internal validation protocol was implemented that was designed following the recommendations provided by “European Community Commission Decision No. 657/2002” [43] and the VICH GL49 “Guidance for Industry” document regarding validation of analytical methods used in residue depletion studies [44].

For each AM, six pure drug replicates were analyzed to evaluate RT. For the determination of the detection range, a preliminary estimation of LOD and LOQ was performed to verify the existence of a linear relationship between concentration and instrument response. These values were determined as instrumental LOD and LOQ. Subsequently, the LOD and LOQ for each analyte were determined in a spiked matrix. The criteria for establishing the LOD was to achieve a signal-to-noise ratio greater than 3:1; while a signal-to-noise ratio higher than 10:1 was used to determine the LOQ. Repeatability was determined by analyzing six sets of samples spiked with either 25, 50, and 75 μg kg^−1^ on the same day. The intra-laboratory reproducibility was determined through the analysis of samples on different days and by a different analyst. Blank samples from poultry litter were analyzed to evaluate the specificity of the method. To determine the linearity of the calibration curve, spiked samples were analyzed at different and equidistant concentrations (25, 50, 75, and 100 µg kg^−1^ including zero). The matrix effect was evaluated comparing the response of the analyte in standard solution with its response in a spiked litter sample, all at the same concentration.

### 2.6. Antimicrobial Monitoring in Commercial Flocks

Litter samples from seven commercial flocks located in the Santiago Metropolitan Region, Chile, were collected. For each poultry farm pen, six replicates were obtained from different litter zones. The samples were stored at −20 °C within labeled plastic bags until its subsequent processing, analyte extraction, and chromatographic analysis.

The quantification of AMs in poultry litters was performed using the equation of the line obtained from calibration curves in a matrix. The R^2^ considered was higher than 0.98. 

## 3. Results

### 3.1. Implementation and Optimization of the Analytical Method

The analytical method by Berendsen et al. [45] was optimized for the identification of AMs in poultry litter. The sample volume was reduced and extraction solvent volumes were increased. It was also necessary to filter through Whatman™ glass microfiber paper filters GF/A grade (1.6 µm) (Merck KGaA, Darmstadt, Germany). The increase of solvent and addition of the paper filter was used to improve the cleanliness of the samples, reducing interference that could affect the chromatographic reading and analysis.

### 3.2. Validation of the Analytical Method

The analytical method developed in this research was validated, and the following results were obtained: 

#### 3.2.1. Selectivity and Specificity.

**Figure 2:**
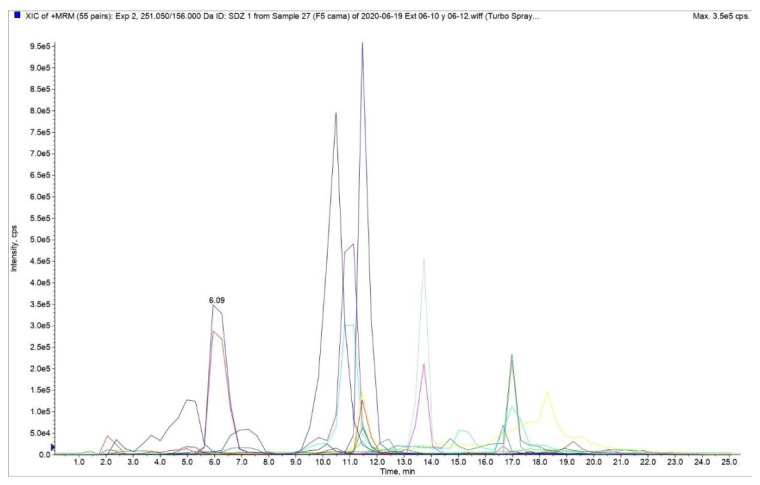
Representative chromatograms of a blank poultry litter sample spiked with 75 µg kg^−1^ of the antimicrobials.

The RT of all analytes remained constant in the six analyses performed and exhibited a relative standard deviation (RSD) of less than 5%. For the tetracycline family the average RT were 10.5, 4.9, 11.7, and 7.3 min for TC, 4-epi-TC, OTC, and 4-epi-OTC, respectively. For the quinolone family the average RT were 11.5, 11.1, and 16.9 min for EFX, CFX, and FLU, respectively. For the sulphonamide family the average RT was 13.7 min for SCP and 6.3 min for SDZ. Finally, the average RT for the I.S. were 11.5, 12.4, and 10.1 min for EFX D5, SMZ 13C6, and TC D6, respectively. A representative chromatogram of a blank poultry litter sample spiked at the target level is shown in Figure 2. To exclude the existence of RT interference specific to each analyte, 20 samples (certified as free of AM residues) were analyzed. The results showed that within the analyzed samples there were no signs of RT interference (Figure S1). The matrix effect was higher than zero occurring loss in response. To minimize this effect, we quantify with calibration curves in matrix.

#### 3.2.2. Detection Range

The LOD and LOQ in spiked poultry litter are shown for each analyte in Table 3. For validation of these parameters, nine samples were spiked at 50 µg kg^−1^. The RSD determined from the analysis of these replicates was less than 25%. The LOQ values were between 26.852 and 62.582 µg kg^−1^ and complied with the minimum signal-to-noise ratio of 10:1. From these concentrations, experimental samples were reliably and accurately quantified. The instrumental LOD and LOQ are shown in Table S3. 

#### 3.2.3. Linearity of Calibration Curves

Different AM concentration levels (25, 50, 75, and 100 µg kg^−1^) were used for the creation of the calibration curves. For all analytes, the coefficients of determination (R^2^) were higher than 0.98 (Table 3), and the RSD values were lower than 25%. The linearity of the curve did not contain significant variations that could affect the robustness of the analytical result. 

#### 3.2.4. Recovery and Precision

Recovery rates were calculated for each analyte based on target samples that were spiked at 25, 50, and 75 µg kg^−1^. All analytes exhibited recovery rates ranging from 93 to 108%. The precision of the HPLC–MS/MS method was measured by intra-laboratory reproducibility and repeatability. The RSD of the intra-laboratory reproducibility was lower than 25%. The RSD for repeatability was less than those observed for intra-laboratory reproducibility (Table 4).

### 3.3. Analysis of Antimicrobial Concentrations in Commercial Flocks Litter

The application of the analytical method for detection and quantification of veterinary pharmaceuticals in litter collected from poultry farms showed that AMs residues were present at means concentrations ranging from 20 to 10,364 μg kg^−1^. The AMs identified were EFX, CFX, SCP, OTC, and TC. In two out of seven poultry litter samples, no residues of any of the AMs studied were detected. The quantification of AMs is shown in Table 5.

## 4. Discussion

Poultry droppings, through poultry litter, are commonly used to improve soil fertility worldwide. Using poultry litter is an economical way to dispose of the waste products generated in substantial quantities in the poultry industry [46]. Global poultry meat production increased from 9 to 122 million tons between 1961 and 2017 [47] and the amount of poultry litter generated by poultry in a production cycle of 42 days of life varies from 1.5 to 5.7 kg of litter per bird [12]. There is evidence indicating that poultry litter does not meet the minimum standards for application as organic fertilizer, mainly due to the presence of contaminants such as AM residues, pathogens, AM resistance genes, heavy metals, etc. Despite this, poultry litter continues to be used and there are no regulations regarding its use as agricultural fertilizer [21].

There is a growing concern for the presence of AM residues, and hence, the persistence and risks of these emerging contaminants have been investigated in both poultry droppings and litter [16,48,49,50,51,52,53]. The primary concern is the continuous pressure on bacteria resulting in the selection of microorganisms with genes encoding resistance to AMs [35]. Therefore, monitoring the use of AMs in both feed and poultry droppings has been described as a key strategy to control the emerging problem of AMs resistance [4]. 

The present research was aimed at developing a noninvasive, efficient, and effective method to sensitively and affordably monitor the presence of AMs in poultry litter prior to its use as an agricultural fertilizer. Although there was an important multiresidue method developed by Furtula et al. [15] to identify different residues of AMs by HLPC–MS/MS in poultry litter, it was mainly limited to the detection of polyether ionophores (e.g., monensin, salinomycin, and narasin). Our method has the advantage of detecting different AMs belonging to the tetracyclines, sulphonamides, and quinolones families. These families of AMs are described by the OIE as some of the most used families worldwide in animals [54,55] and are widely used in the poultry industry [4,5]. Moreover, unlike other methods [56], ours allows the simultaneous analysis of AMs in both their parent molecule and the metabolite forms (epimers or isomers) [9]. 

The multiresidual method developed by Berendsen et al. [45], which detects AMs in porcine and bovine feces, provides us the basis for this research. The present method, modified for poultry litter, was optimized by decreasing the gradient, using different solid-phase extraction columns, increasing the solvents (McIlvaine–EDTA buffer and acetonitrile), using microfiber paper filters, and adding additional steps such as the grinding of the raw sample to decrease the poultry litter diameter. These modifications improved the samples cleanup, which reduced the presence of impurities that could interfere with the chromatographic analysis. The optimized analytical method was able to detect and quantify the concentration of all analytes evaluated in poultry litter in a selective, accurate, and reliable manner. All parameters determined during the validation process of the HPLC–MS/MS method comply with the acceptance criteria according to 657/2002/EC [43] and the VICH GL49 [44].

The analysis of samples obtained from commercial flocks confirmed the adequacy of this method. In this sense, high concentrations of EFX and OTC detected in litter samples may be associated with the use of these AMs, considering the number of pharmacological formulations registered for use in poultry [6]. The highest concentrations of AMs residues in field samples from poultry farms correspond to EFX. This is consistent with previous research performed in Brazil, which confirms EFX as the quinolone most often detected, and with the highest mean concentrations in poultry litters samples [16]. Results from commercial flocks show that antimicrobials used in poultry production might represent one potential source of dissemination of AMs residues into the environment. Accordingly, we developed a simple and affordable method for detecting and quantifying antimicrobials in poultry litter through HPLC–MS/MS. This can be considered to be a significant step forward for future AMs residues monitoring programs in this poultry by-product.

## 5. Conclusions

The analytical method developed for the simultaneous detection and quantification of AMs was validated to ensure the residues of tetracycline, 4-epi-tetracycline, oxytetracycline, 4-epi-oxytetracycline, enrofloxacin, ciprofloxacin, flumequine, sulfachloropyridazine, and sulfadiazine can be accurately and reliably detected in poultry litter. Additionally, the method developed here was successfully tested to detect and quantify antimicrobials residues in litter samples obtained from commercial flocks.

The method described could be employed as an affordable tool for monitoring and understanding the presence and persistence of antimicrobials in poultry litter. Effective monitoring can help to mitigate effects on human, animal, and environmental health that may arise from the use of antimicrobial agents. 

## Figures and Tables

**Table 1:** Liquid chromatographic pump gradient.

Total Time (min)	Flow Rate(µL/min)	A (%)	B (%)
0.00	200	85.0	15.0
5.00	200	85.0	15.0
5.10	200	60.0	40.0
10.00	200	60.0	40.0
10.10	200	10.0	90.0
15.00	200	10.0	90.0
16.00	200	85.0	15.0
25.00	200	85.0	15.0

**Table 2:** Operation parameters of the MS/MS detector.

Parameters	Analytical Conditions
Ionization	Electrospray ionization (ESI)
Temperature	550 °C
Curtain gas	30 psi
Collision gas	10 psi
Ion spray voltage	4500 V
Ion source gas 1	60 psi
Ion source gas 2	80 psi

**Table 3:** Linearity of calibration curves and detection ranges in poultry litter.

Analyte	Linearity (R^2^) ^10^	R^2^ SD ^11^	R^2^ RSD% ^12^	LOD ^13^ (µg kg^−1^)	LOQ ^14^ (µg kg^−1^)
TC ^1^	0.985	0.004	0.373	10.711	32.133
4-epi-TC ^2^	0.977	0.001	0.070	8.951	26.852
OTC ^3^	0.979	0.003	0.347	11.487	34.460
4-epi-OTC ^4^	0.977	0.002	0.179	9.379	28.138
EFX ^5^	0.986	0.003	0.331	13.626	40.879
CFX ^6^	0.980	0.011	1.127	20.861	62.582
FLU ^7^	0.980	0.005	0.555	11.729	35.188
SCP ^8^	0.982	0.006	0.561	9.191	27.574
SDZ ^9^	0.977	0.011	1.171	11.705	35.116

^1^ TC: tetracycline; ^2^ 4-epi-TC: 4-epimer-tetracycline; ^3^ OTC: oxytetracycline; ^4^ 4-epi-OTC: 4-epimer-oxytetracycline; ^5^ EFX: enrofloxacin; ^6^ CFX: ciprofloxacin; ^7^ FLU: flumequine; ^8^ SCP: sulfachloropyridazine; ^9^ SDZ: sulfadiazine; ^10^ average R^2^: coefficient of determination of 3 calibration curves in matrix spiked with 25, 50, 75, and 100 µg kg^−1^ of antimicrobials, including zero; ^11^ standard deviation; ^12^ relative standard deviation; ^13^ limit of detection in matrix; ^14^ limit of quantification in matrix.

**Table 4:** Repeatability, intra-laboratory reproducibility, and antimicrobial recovery in poultry litter.

Analyte	Work Concentration (µg kg^−1^)	Repeatability RSD ^10^ (%)	Reproducibility RSD (%)	Average Recovery (%)
TC ^1^	25	1.78	5.17	94.6
	50	1.69	4.64	105.4
	75	0.58	1.66	98.2
4-epi-TC ^2^	25	2.51	9.85	91.7
	50	2.82	8.34	108.3
	75	0.87	3.10	97.2
OTC ^3^	25	3.94	6.38	92.4
	50	3.72	5.48	107.6
	75	1.29	2.01	97.5
4-epi-OTC ^4^	25	5.05	7.13	95.8
	50	4.65	6.57	104.2
	75	1.64	2.31	98.6
EFX ^5^	25	4.96	10.64	95.4
	50	5.61	9.71	104.6
	75	1.72	3.44	98.5
CFX ^6^	25	7.58	9.60	95.8
	50	8.23	8.83	104.2
	75	2.60	3.11	98.6
FLU ^7^	25	3.68	6.91	93.7
	50	3.62	6.09	106.3
	75	1.22	2.20	97.9
SCP ^8^	25	3.31	4.91	93.0
	50	3.29	4.27	107.0
	75	1.10	1.56	97.7
SDZ ^9^	25	1.59	10.01	95.6
	50	1.92	9.17	104.4
	75	0.56	3.24	98.5

^1^ TC: tetracycline; ^2^ 4-epi-TC: 4-epimer-tetracycline; ^3^ OTC: oxytetracycline; ^4^ 4-epi-OTC: 4-epimer-oxitetracycline; ^5^ EFX: enrofloxacin; ^6^ CFX: ciprofloxacin; ^7^ FLU: flumequine; ^8^ SCP: sulfachloropyridazine; ^9^ SDZ: sulfadiazine; ^10^ relative standard deviation.

**Table 5:** Antimicrobial residues in poultry litter samples from commercial poultry farms.

Poultry Litter Sample	TC ^1^ + 4-epi-TC ^2^ (µg kg^−1^)	OTC ^3^ + 4-epi-OTC ^4^ (µg kg^−1^)	EFX ^5^ + CFX ^6^ (µg kg^−1^)	FLU ^7^ (µg kg^−1^)	SCP ^8^ (µg kg^−1^)	SDZ ^9^ (µg kg^−1^)
Farm 1	nd	nd	20.73 ± 10 ^10^	nd	nd	nd
Farm 2	nd	nd	nd	nd	nd	nd
Farm 3	nd	nd	nd	nd	179.99 ± 83	nd
Farm 4	nd	nd	24,307.11 ± 10	nd	nd	nd
Farm 5	nd	nd	nd	nd	nd	nd
Farm 6	nd	66.43 ± 53	nd	nd	nd	nd
Farm 7	322.10 ± 229	10,364.41 ± 6791	nd	nd	nd	nd

^1^ TC: tetracycline; ^2^ 4-epi-TC: 4-epimer-tetracycline; ^3^ OTC: oxytetracycline; ^4^ 4-epi-OTC: 4-epimer-oxitetracycline; ^5^ EFX: enrofloxacin; ^6^ CFX: ciprofloxacin; ^7^ FLU: flumequine; ^8^ SCP: sulfachloropyridazine; ^9^ SDZ: sulfadiazine; ^10^ standard deviation; nd: not detected.

## Data Availability

The data presented in this research are available in the article and Supplementary Material.

## Supplementary Materials

The following are available online at https://www.mdpi.com/article/10.3390/ani11051399/s1, Figure S1: Representative chromatograms from (1) a pure standard solution injection and (2) a blank poultry litter sample of (a) tetracycline, (b) 4-epimer-tetracycline, (c) oxytetracycline, (d) 4-epimer-oxytetracycline, (e) enrofloxacin, (f) ciprofloxacin, (g) flumequine, (h) sulfachloropyridazine, (i) sulfadiazine, Table S1: Scans per peak, Table S2: Mass of precursor and fragment ions, and specific mass spectrometry conditions, Table S3: Instrumental Limit of detection and Limit of quantification.
