# Supplementary material for: Detection of Antimicrobial Residues in Poultry Litter: Monitoring a Risk through a Selective and Sensitive HPLC–MS/MS Method
Source: Animals (Basel). 2021 May 14;11(5):1399. doi: 10.3390/ani11051399 (PMC8156041; doi:10.3390/ani11051399)
Supplement: Supplementary file 1 [file animals-11-01399-s001.zip › animals-1185636-SI.pdf]

*animals*

MDPI

*Supplementary Materials*

# Detection of antimicrobial residues in poultry litter: monitoring a risk through a selective and sensitive HPLC-MS/MS method

**Karina Yévenes, Ekaterina Pokrant, Lina Trincado, Lisette Lapierre, Nicolás Galarce, Betty San Martín, Aldo Maddaleno, Héctor Hidalgo and Javiera Cornejo**

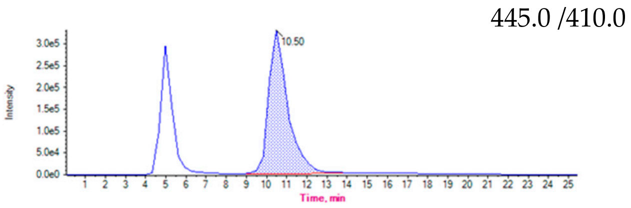


(a1)

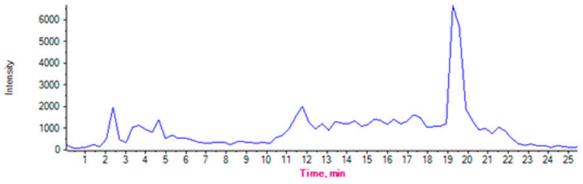


(a2)

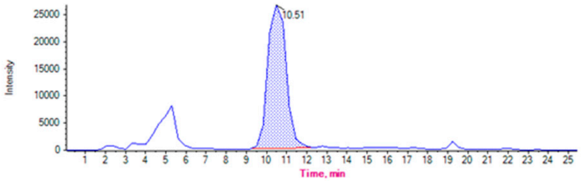


(a3)

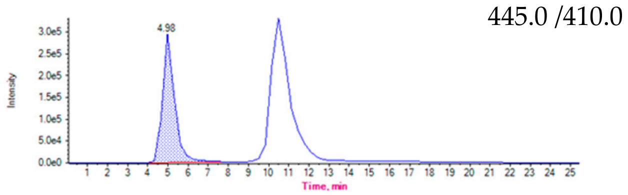


(b1)

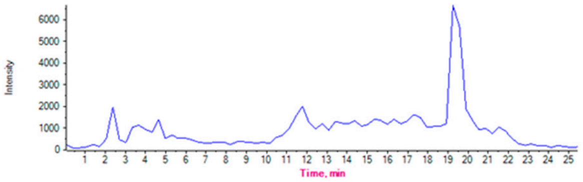


(b2)

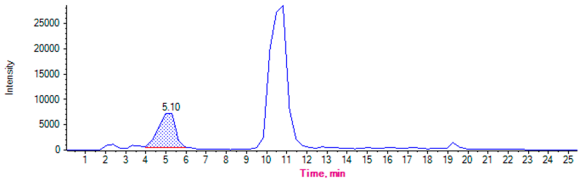


(b3)

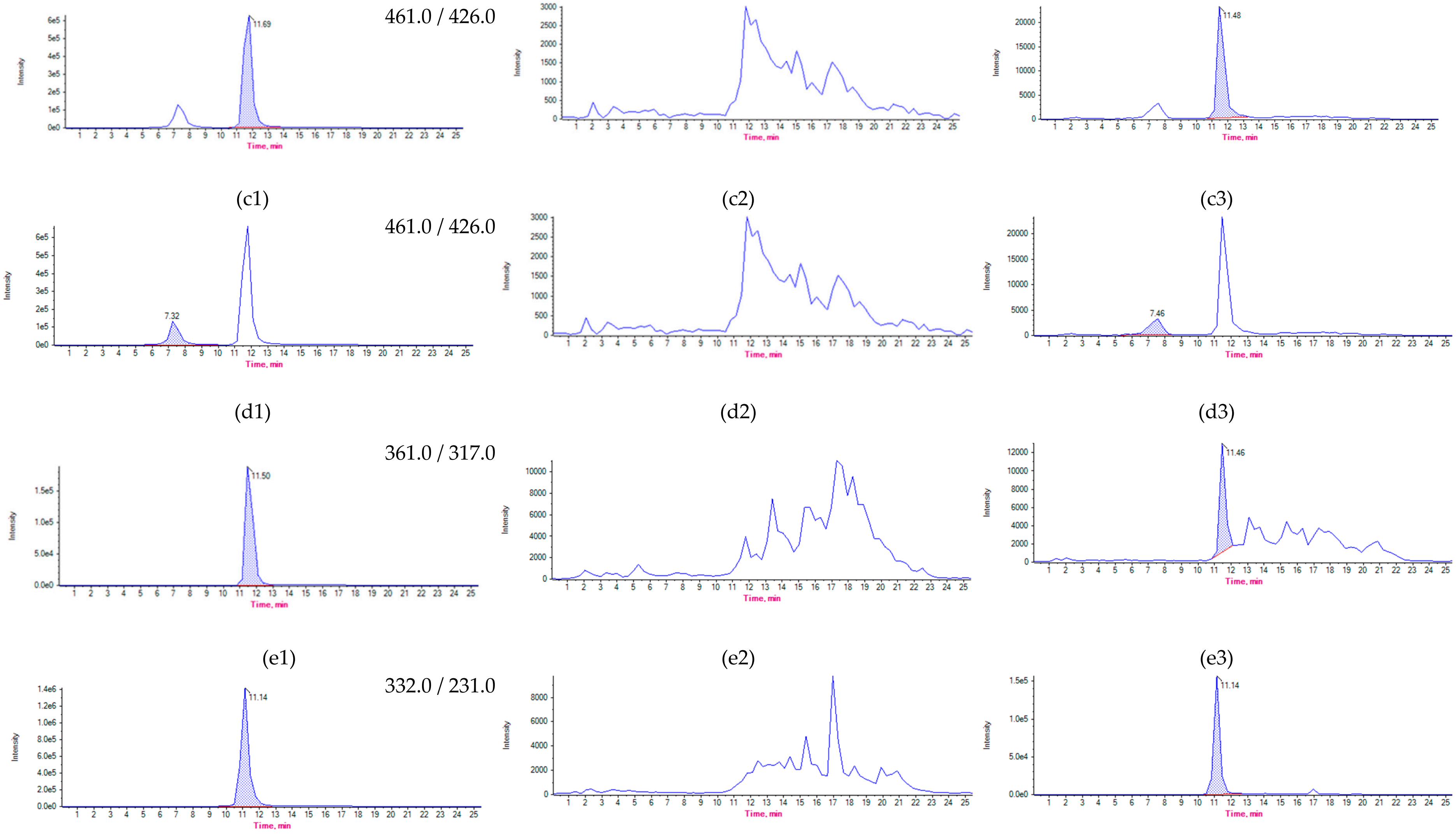


(c1) (c2) (c3)

(d1) (d2) (d3)

(e1) (e2) (e3)

(f1) (f2) (f3)

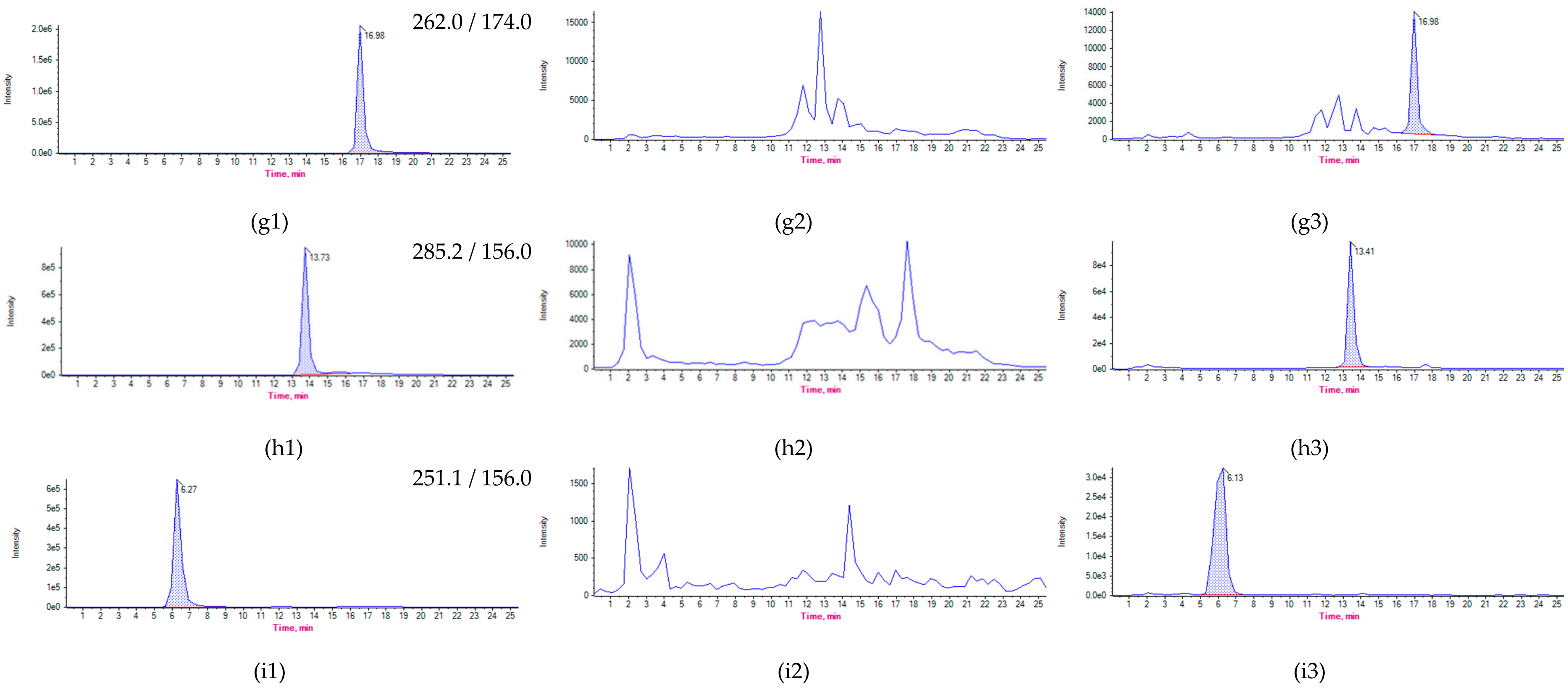


**Figure S1.** Representative chromatograms from (1) a pure standard solution injection (50 ng mL-1), (2) a blank poultry litter sample and (3) poultry litter sample spiked to 25 ug k-1 of (a) tetracycline, (b) 4-epimer-tetracycline, (c) Oxitetracycline, (d) 4-epimer-oxitetracycline, (e) Enrofloxacin, (f) Ciprofloxacin, (g) Flumequine, (h) Sulfachloropyridazine, (i) Sulfadiazine.

**Table S1.** Scans per peak.

| Analytes | Time (msec) |
|---|---|
| Tetracycline / 4-epimer-Tetracycline | 100.0 |
| Oxytetracycline / 4-epimer-Oxytetracycline | 100.0 |
| Enrofloxacin | 280.0 |
| Ciprofloxacin | 280.0 |
| Flumequine | 280.0 |
| Sulfachloropyridazine | 280.0 |
| Sulfadiazine | 200.0 |
| Sulfamethazine-phenyl-13C6 | 280.0 |
| Enrofloxacin-D5 | 280.0 |
| Tetracycline-D6 | 250.0 |

**Table S2.** Mass of precursor and fragment ions, and specific mass spectrometry conditions.

| Analytes | Precursor Ion (m/z) | Fragment Ions (m/z) | DP [3] (V) | EP [4] (V) | CE [5] (V) | CXP [6] (V) |
|---|---|---|---|---|---|---|
| Tetracycline / 4-epimer-Tetracycline | 445.0 | 410.0[1] | 77.0 | 10.0 | 28.0 | 24.0 |
| | | 392.0 | 69.0 | 10.0<br>0 | 38.0 | 23.0 |
| Oxytetracycline / 4-epimer-Oxytetracycline | 461.0 | 426.0[1] | 72.0 | 10.0 | 28.0 | 25.0 |
| | | 381.0 | 73.0 | 10.0 | 36.0 | 22.0 |
| Enrofloxacin | 361.0 | 343.0 | 50.0 | 13.0 | 23.0 | 8.0 |
| | | 317.0[1] | 50.0 | 13.0 | 28.0 | 20.0 |
| Ciprofloxacin | 332.0 | 231.0[1] | 56.0 | 4.5 | 47.0 | 4.0 |
| | | 314.0 | 56.0 | 4.5 | 28.0 | 4.0 |
| Flumequine | 262.0 | 244.0 | 20.0 | 5.0 | 10.0 | 13.0 |
| | | 174.0[1] | 20.0 | 5.0 | 50.0 | 7.0 |
| Sulfachloropyridazine | 285.2 | 156.0[1] | 61.0 | 10.0 | 21.0 | 12.0 |
| | | 108.1 | 61.0 | 10.0 | 31.0 | 8.0 |
| Sulfadiazine | 251.1 | 156.0[1]<br>108.0 | 46.0 | 4.0 | 19.0 | 4.0 |
| | | 108.0 | 46.0 | 4.0 | 31.0 | 4.0 |
| Sulfamethazine-phenyl-13C6[2] | 285.0 | 124.1 | 71.0 | 10.0 | 31.0 | 12.0 |
| Enrofloxacin-D5[2] | 365.0 | 321.0 | 50.0 | 7.0 | 23.0 | 8.0 |
| Tetracycline-D6[2] | 451.0 | 160.0 | 34.0 | 10.0 | 25.0 | 30.0 |

[1] Quantifier ion; [2] Internal Standard; [3] Declustering potential, [4] Entrance potential, [5] Collision energy, [6] Collision cell exit potential.

**Table S3.** Instrumental Limit of detection and Limit of quantification.

| **Analyte** | **ILOD [1] (µg kg$^{-1}$)** | **ILOQ [2] (µg kg$^{-1}$)** |
|---|---|---|
| Enrofloxacin | 2.0 | 6.7 |
| Ciprofloxacin | 3.1 | 10.4 |
| Flumequine | 2.9 | 9.7 |
| Sulfachloropyridazine | 3.0 | 10.1 |
| Sulfadiazine | 2.8 | 9.5 |
| Tetracycline | 3.4 | 11.4 |
| 4-epi-Tetracycline | 2.9 | 9.7 |
| Oxytetracycline | 2.7 | 8.9 |
| 4-epi-Oxytetracycline | 2.3 | 7.8 |
| Chlortetracycline | 3.3 | 11.1 |
| 4-epi-Chlortetracycline | 2.9 | 9.6 |

[1]Instrumental Limit of detection; [2]Instrumental Limit of quantification.
